# Cisplatin anti-tumour potentiation by tirapazamine results from a hypoxia-dependent cellular sensitization to cisplatin

**DOI:** 10.1038/sj.bjc.6690492

**Published:** 1999-06

**Authors:** M S Kovacs, D J Hocking, J W Evans, B G Siim, B G Wouters, J M Brown

**Affiliations:** Cancer Biology Research Laboratory, Department of Radiation Oncology, GK103, Stanford, CA, 94305, USA

**Keywords:** tirapazamine, cisplatin, hypoxia, DNA interstrand cross-links, nucleotide excision repair

## Abstract

Tirapazamine (TPZ) is a new anticancer drug that is activated specifically at the low oxygen level typically found in solid tumours. It exhibits preferential cytotoxicity towards hypoxic cells and has been shown in preclinical studies with transplanted tumours and in phase II and III clinical trials to potentiate the anti-tumour efficacy of cisplatin without increasing its systemic toxicity. At present, the mechanism for this potentiation is unknown. Here we show that there is a schedule-dependent enhancement of cisplatin cytotoxicity by TPZ for cells in vitro that is similar to that seen with transplanted murine tumours. This cisplatin potentiation depends on the TPZ exposure being at oxygen concentrations below 1%, which are typical of many cells in tumours but not in normal tissues. Also, the interaction between TPZ and cisplatin does not occur in cells mutant in ERCC4, a protein essential for repair of DNA interstrand cross-links. Incubation of the cells with TPZ under hypoxia prior to cisplatin treatment increases cisplatin-induced DNA interstrand cross-links with kinetics suggesting that TPZ inhibits or delays repair of the DNA cross-links. In conclusion, we show that the tumour-specific potentiation of cisplatin cytotoxicity is likely the result of an interaction between TPZ and cisplatin at the cellular level that requires the low oxygen levels typical of those in solid tumours. The mechanism of the interaction appears to be through a potentiation of cisplatin-induced DNA interstrand cross-links, possibly as a result of a diminished or delayed repair of these lesions © 1999 Cancer Research Campaign


*British Journal of Cancer* (1999) **80**(8), 1245–1251
© 1999 Cancer Research Campaign
Article no. bjoc.1998.0492

### Cisplatin anti-tumour potentiation by tirapazamine results from a hypoxia-dependent cellular sensitization to cisplatin

**MS Kovacs, DJ Hocking, JW Evans, BG Siim*, BG Wouters and JM Brown**

Cancer Biology Research Laboratory, GK103, Department of Radiation Oncology, Stanford University School of Medicine, Stanford, CA 94305, USA

**Summary** Tirapazamine (TPZ) is a new anticancer drug that is activated specifically at the low oxygen level typically found in solid tumours. It exhibits preferential cytotoxicity towards hypoxic cells and has been shown in preclinical studies with transplanted tumours and in phase II and III clinical trials to potentiate the anti-tumour efficacy of cisplatin without increasing its systemic toxicity. At present, the mechanism for this potentiation is unknown. Here we show that there is a schedule-dependent enhancement of cisplatin cytotoxicity by TPZ for cells in vitro that is similar to that seen with transplanted murine tumours. This cisplatin potentiation depends on the TPZ exposure being at oxygen concentrations below 1%, which are typical of many cells in tumours but not in normal tissues. Also, the interaction between TPZ and cisplatin does not occur in cells mutant in ERCC4, a protein essential for repair of DNA interstrand cross-links. Incubation of the cells with TPZ under hypoxia prior to cisplatin treatment increases cisplatin-induced DNA interstrand cross-links with kinetics suggesting that TPZ inhibits or delays repair of the DNA cross-links. In conclusion, we show that the tumour-specific potentiation of cisplatin cytotoxicity is likely the result of an interaction between TPZ and cisplatin at the cellular level that requires the low oxygen levels typical of those in solid tumours. The mechanism of the interaction appears to be through a potentiation of cisplatin-induced DNA interstrand cross-links, possibly as a result of a diminished or delayed repair of these lesions

**Keywords:** tirapazamine; cisplatin; hypoxia; DNA interstrand cross-links; nucleotide excision repair

Tirapazamine (3-amino-1,2,4-benzotriazine 1,4-dioxide: SR 4233) (TPZ) is a new anticancer drug that is activated in hypoxic cells to a highly damaging free radical (Brown, 1993), resulting in selective cytotoxicity towards hypoxic cells (Zeman et al, 1986; Brown, 1993). Because of this preferential toxicity to hypoxic cells, TPZ was originally developed as an adjunct to radiation therapy, since there is compelling evidence that hypoxic cells in tumours lead to failure of local control by radiotherapy (Gatenby et al, 1988; Nordsmark et al, 1996; Brizel et al, 1997). In support of this use, preclinical experiments have shown that the addition of TPZ to fractionated irradiation leads to significantly greater tumour cell kill without enhancing radiation damage to normal tissues (Brown and Lemmon, 1990, 1991). These studies have led to clinical trials of TPZ combined with radiotherapy (Lee et al, 1997).

However, in addition to its potentiation of fractionated radiation, TPZ also produces a schedule-dependent enhancement of the anti-tumour activity of cisplatin and carboplatin in experimental tumours (Dorie and Brown, 1993, 1997; Durand, 1994; Mangold et al, 1997; Siemann and Hinchman, 1998). The potentiation of tumour cell kill by TPZ when added to these platinum-containing drugs is significant for cancer therapy as the drug does not enhance the systemic toxicity of either cisplatin or carboplatin (Dorie and Brown, 1993, 1997; Siemann and Hinchman, 1998). These studies have led to clinical testing of TPZ combined with cisplatin with a variety of tumours (Rodriguez et al, 1996; Graham et al, 1997; Miller et al, 1997; Treat et al, 1997). A recently completed phase III study of TPZ combined with cisplatin for stage IIIB and IV non-small-cell lung cancer has shown a doubling of overall response rates and a significant survival advantage compared to cisplatin alone (von Pawel and von Roemeling, 1998).

Despite these promising preclinical and clinical results, the mechanism by which TPZ potentiates the anti-tumour effectiveness of platinum-based anticancer therapies is unknown. An obvious possibility is that since TPZ selectively kills hypoxic cells in tumours (Durand, 1994; Kim and Brown, 1994), and these cells are resistant to killing by cisplatin (Grau and Overgaard, 1988), there should be complementary cell kill between the two drugs. Indeed, evidence for such complementarity of cell killing between cisplatin and TPZ has been obtained with human tumour xenografts by Durand (1994). However, this cannot account for all of the activity, since we have obtained preliminary data that pretreatment of hypoxic cells in vitro can sensitize them to a subsequent cell killing by cisplatin (Dorie and Brown, 1993). Also, Durand has demonstrated in a xenograft model that the combined killing of TPZ plus cisplatin is greater than that predicted on the basis of additive cytotoxicities (Durand, 1994). Of particular interest in the latter data is that potentiation of cisplatin toxicity occurred even in the cells closest to blood vessels, suggesting that more than the hypoxic cells might be sensitized to cisplatin by TPZ. However, this is not consistent with the conclusion from other studies that the damaging species from TPZ is a radical with a very short, subcellular diffusion distance (Brown, 1993; Evans et al, 1998).

*Received 20 October 1998*
*Revised 17 December 1998*
*Accepted 18 December 1998*

*Correspondence to:* JM Brown

*Present address: Department of Pathology, University of Auckland School of Medicine, Auckland, New Zealand.

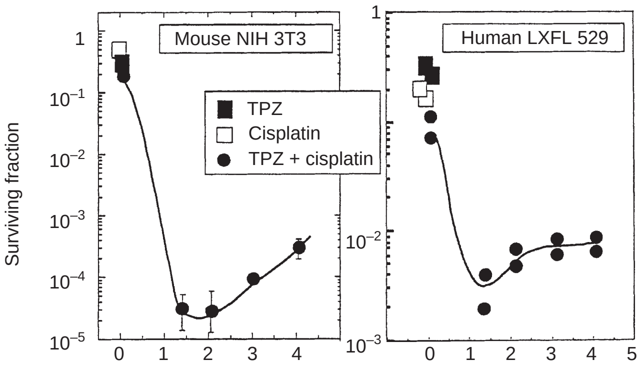

**Figure 1** Effect on clonogenic survival of combining a 1-h exposure to TPZ (20 μM) under hypoxia with a 1-h exposure to cisplatin (20 μM) on mouse NIH 3T3 cells or human LXFL 529 non-small-cell lung cancer cells. The two drugs were given either at the same time (0 h between TPZ and cisplatin) or the cisplatin exposure was given at varying times after the TPZ exposure. Pooled data from two experiments for each cell line (means and s.e.m.)

The present studies were therefore undertaken to explore in more depth the nature of the interaction between TPZ and cisplatin, to examine its oxygen dependence, and to attempt to determine the mechanism for the interaction. We show that much, if not all, of the potentiation of the antitumour effects in vivo can be explained by a cellular interaction that depends on TPZ metabolism under hypoxia. Further, the potentiation is associated with an increase in DNA interstrand cross-links following cisplatin exposure that may be related to an inhibition of, or delay in, the repair of these lesions.

### MATERIALS AND METHODS

### Cell lines

Cell lines were maintained as monolayer cultures in vitro in a humidified atmosphere of 95% air and 5% carbon dioxide at 37°C. The NIH 3T3 immortalized mouse fibroblast line was obtained from the American Type Culture Collection (Rockville, MD, USA) and was cultured in Dulbecco's modified Eagles medium (DMEM) with 10% fetal bovine serum (FBS, Life Technologies, Inc., Gaithersburg, MD, USA). The AA8 and UV41 Chinese hamster ovary cell lines were kindly provided by Dr Larry Thompson (Lawrence Livermore National Laboratory, Livermore, CA, USA). UV41 is a mutant cell line derived from the parental AA8 line that is hypersensitive to DNA cross-linking agents and defective in the ERCC4/XPF gene (Andersson et al, 1996). These two lines were cultured in DMEM (Life Technologies, Inc.) with 10% FBS. The human non-small-lung cancer cell line, LXFL 529, was obtained from the National Cancer Institute, NIH, and is one of the cell lines in the panel of 60 human cell lines used to screen for potential new anticancer compounds. This cell line was cultured in RPMI-1640 medium with 10% FBS.

### Drug exposures and clonogenic assay

For both the colony forming and alkaline elution experiments, cells were plated in 60-mm glass dishes 1–2 days prior to the experiment at $2 \times 10^5$–$1 \times 10^6$ cells per dish. TPZ and cisplatin were made up into solution immediately before the experiment and added to the cells in complete medium. Hypoxia (less than 200 ppm $O_2$) was achieved by exposing the glass dishes in prewarmed, air-tight aluminium jigs to a series of five rapid evacuations and flushings with 95% nitrogen + 5% carbon dioxide in a 37°C water bath on a shaking platform (controls were flushed with 95% air + 5% carbon dioxide). After the fifth evacuation and flushing, the platform (with water bath and jigs) was shaken for 5 min, then one more evacuation and flushing was performed, and the jigs were transferred to a shaker in a 37°C incubator for the remainder of the 1-h drug exposure. Levels of oxygenation between 200 ppm and air were achieved by varying the degree and number of evacuations. The oxygen concentrations in the medium and gas phases was checked using an oxygen electrode (Animas, Phoenixville, PA, USA) in a specially modified aluminium jig that allowed monitoring of both gas and liquid phases. Following the 1 h exposure to TPZ, the aluminium vessels were opened, the TPZ washed off the cells and cisplatin added for 1 h in air at varying times later. At the end of the drug treatment period the cisplatin-containing media was removed, the cell monolayers were washed twice in medium, and either returned to the 37°C incubator for varying times in the alkaline elution experiments, or trypsinized, and plated for clonogenic survival in plastic Petri dishes. The plating efficiency of all cells used was more than 60%. Ten to 14 days later the dishes were stained with crystal violet (0.25% in 95% ethanol) and colonies containing more than 50 cells were counted.

### Measurement of DNA interstrand cross-links by alkaline filter elution

Alkaline elution to measure DNA single-strand breaks and DNA interstrand cross-links was performed essentially as published (Ewig and Kohn, 1978). Briefly, exponentially growing cells were radiolabelled with 2-$^{14}$C thymidine (0.01 μCi ml$^{-1}$) (Amersham, Cleveland, OH, USA) for 24 h, followed by a 4-h chase period. They were then treated with TPZ and/or cisplatin as described above and, after an appropriate incubation period to develop and repair cross-links, were trypsinized, resuspended in ice-cold phosphate-buffered saline (PBS) and half of the cells irradiated with 4 Gy at 4°C. Irradiated and/or drug-treated cells were loaded onto a 2-μm polycarbonate filter, lysed with 2% sodium dodecyl sulphate (SDS), 0.1 M glycine, 25 mM EDTA, pH 10.0 containing 1 mg ml$^{-1}$ proteinase K (Boehringer Mannheim, Indianapolis, IN, USA) and incubated for 1 h at room temperature. DNA was eluted from the filter by alkaline buffer (0.1% SDS, 25 mM EDTA-free acid, and sufficient tetrapropylammonium hydroxide to yield pH 12.2) at a flow rate of 0.033 ml min$^{-1}$ for 12 h. Radioactivity from the eluted fractions, or from the DNA retained on the filter, was measured on a Beckman LS6000IC (Fullerton, CA, USA). Data were analysed by plotting the log of the fraction of labelled DNA retained on the filter versus time. The cross-link index was calculated from the ratio of the radioactivity retained on the filter after 10.5 h of elution for the radiation plus cisplatin-treated groups divided by the corresponding radiation only group essentially as described (Ewig and Kohn, 1978).

### RESULTS

### Time-dependent potentiation of cisplatin cell kill

We first measured cell killing of the combination of TPZ (1-h exposure under hypoxia) with cisplatin (1-h exposure) as a function of

 © 1999 Cancer Research Campaign

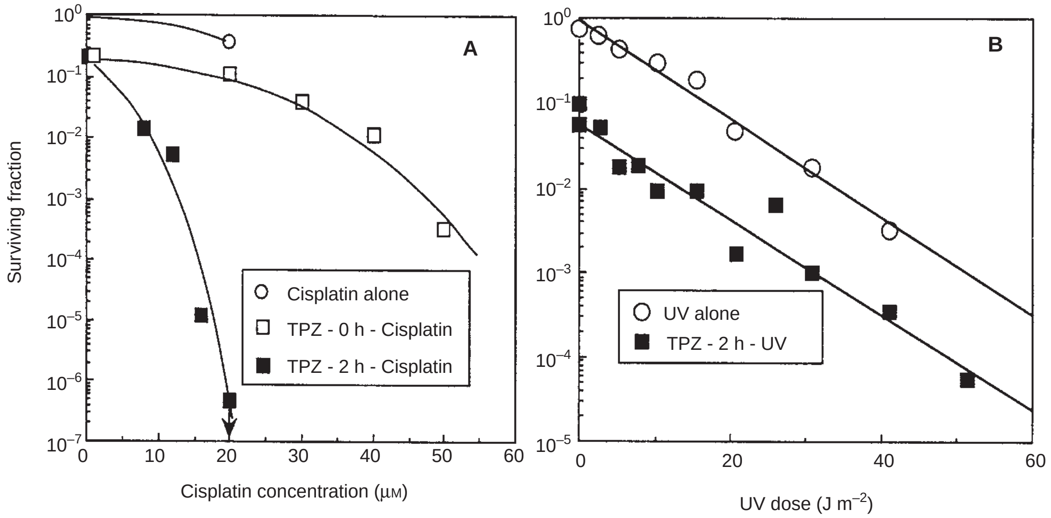

**Figure 2** Clonogenic survival of NIH 3T3 cells treated with a 1-h exposure to TPZ (20 μM) combined either with cisplatin (**A**) or with UV irradiation (**B**). In panel **A** the combination of TPZ with cisplatin was performed either with the two drugs given together for 1 h (TPZ – 0 h – CisPt) or with the 1-h cisplatin exposure started 2 h after the beginning of the 1-h TPZ exposure under hypoxia (TPZ – 2 h – CisPt). In panel **B** the UV exposure was given either alone (2 h following the start of a 1-h exposure of the cells under hypoxia alone), or 2 h after the start of a 1-h exposure to TPZ under hypoxia. The data show the results of single experiments time between the two exposures using minimally toxic doses of each agent (less than 1 log of cell kill). As can be seen in Figure 1, the combination of TPZ and cisplatin given at the same time under hypoxic conditions produces approximately additive cell kill. However, a marked potentiation of cell kill occurs when the TPZ exposure precedes the cisplatin exposure. Note that the kinetics, though not the magnitude, of the potentiation is similar for mouse NIH 3T3 and the LXFL 529 human non-small-cell lung cancer cell lines. In the groups in which cisplatin was given after the TPZ exposure, the cisplatin dose was given under aerobic conditions. However, we also measured survival when the cisplatin treatment was administered under hypoxic conditions. In this case, the sensitivity of the cells to either cisplatin alone or to the combination of TPZ and cisplatin was the same as when cisplatin was administered under aerobic conditions (data not shown). We also saw no influence on the sensitivity of the cells to cisplatin if the TPZ exposure was under aerobic conditions (see later).

To quantitate the extent of the potentiation of cisplatin toxicity by prior exposure of cells to TPZ under hypoxia, we measured complete dose–response curves for cisplatin in cells treated either 2 h prior to, or concurrently with TPZ. As can be seen from Figure 2A, NIH 3T3 cells were sensitized by a dose modification factor

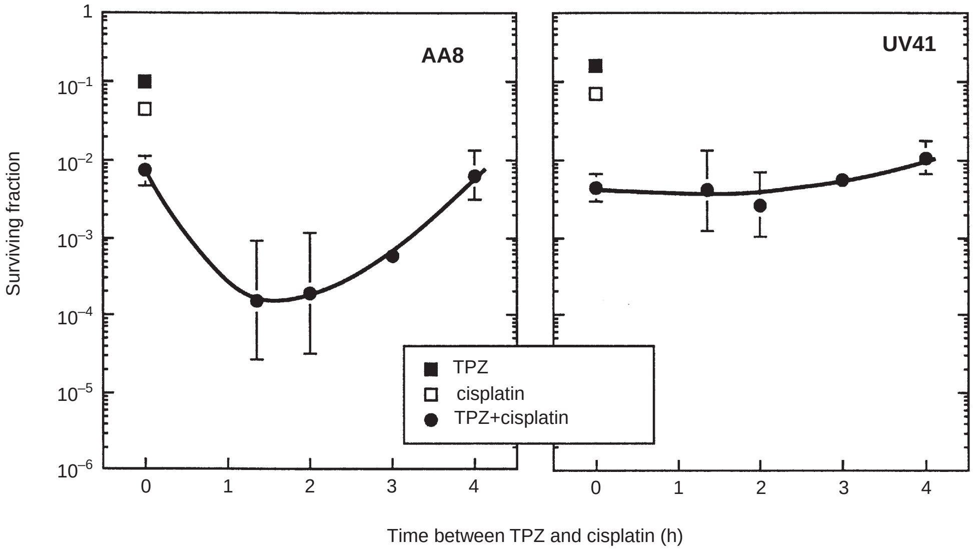

**Figure 3** Effect on clonogenic survival of combining a 1-h exposure to TPZ (18 μM) with cisplatin (20 μM) on hamster AA8 cells or to a 1-h exposure to TPZ (6 μM) with cisplatin (0.3 μM) on the cross-link repair-deficient cell line UV41. In both cases the TPZ and cisplatin were given either at the same time or the cisplatin was given at varying times from 1 to 4 h after the start of the 1-h TPZ exposure under hypoxia. Pooled data from three experiments for each cell line (means and s.e.m.)

*© 1999 Cancer Research Campaign* 

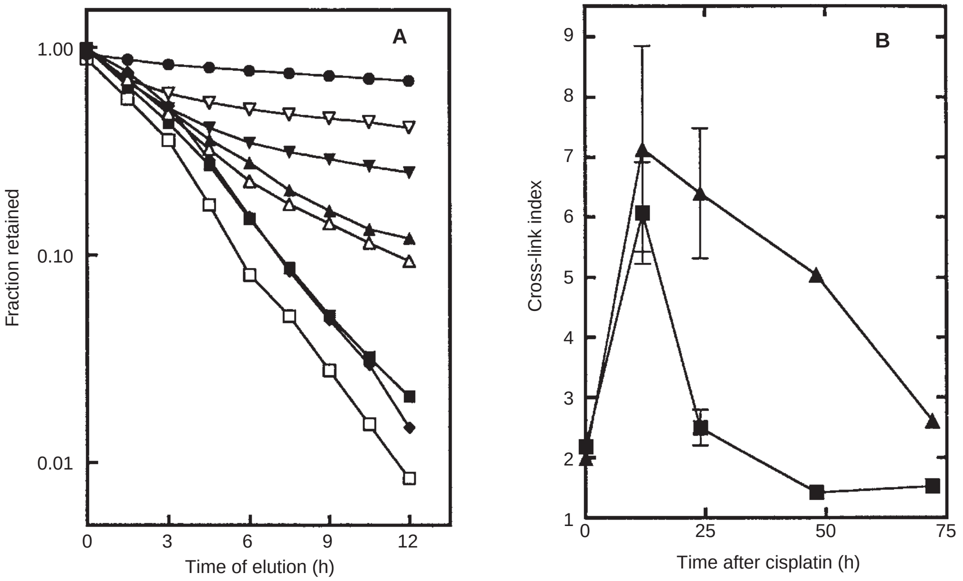

**Figure 4** (**A**) Alkaline filter elution profiles of NIH3T3 cells treated at 48 and 72 h after cisplatin (50 μM) either alone or 1.5 h after the start of a 1-h exposure to TPZ (20 μM) under hypoxia. The cells treated with cisplatin alone were given a 1-h exposure to hypoxia 1.5 h before the cisplatin treatment. All groups except the control untreated group received a radiation dose of 4 Gy on ice immediately prior to cell lysis. The groups are:(●), control untreated cells;▽, TPZ plus cisplatin 48 h; ▼, TPZ plus cisplatin 72 h; ▲, cisplatin alone 72 h; △, cisplatin alone 48 h; ■, TPZ alone (72 h); ◆, 4 Gy alone; □, TPZ alone (48 h). Data from a single experiment. (**B**) Calculated cross-link index for cells treated with cisplatin alone (■) and those treated with TPZ 2 h before cisplatin (▲) as a function of time after the cisplatin treatment. Pooled results of two experiments (means and s.e.m.)

(DMF) of 3.5–4.0 when the TPZ and cisplatin drug exposures were separated by 2 h compared with cells treated with TPZ and cisplatin simultaneously.

Since at least some of the proteins involved in nuclear excision repair (NER), a pathway involved in the repair of damage by UV irradiation, also affect cellular sensitivity to cisplatin (Damia et al, 1996), we determined whether TPZ enhanced the sensitivity of cells to UV irradiation. As Figure 2B shows, the combination of UV given 2 h after a hypoxic exposure to TPZ produced only additive cell kill. This experiment suggests that the interaction with TPZ is not directly through the NER pathway, but rather involves a pathway that is involved specifically in the sensitivity of cells to cisplatin.

### Mechanism of the interaction

Cells deficient in DNA interstrand cross-link repair are hypersensitive to killing by cisplatin and to other agents that kill cells by the formation of DNA interstrand cross-links (Damia et al, 1996). We therefore tested cells deficient in DNA interstrand cross-link repair for the interaction between TPZ and cisplatin. For this we used the hamster cell UV41, which is defective in the gene ERCC4, and which is hypersensitive to killing by mitomycin C and to other agents that produce DNA interstrand cross-links (Jones et al, 1990; Andersson et al, 1996). Figure 3 shows the data for hamster UV41 cells and their wild-type counterpart, AA8 cells. As before, the combination of TPZ with cisplatin produced additive cytotoxicity when the two agents were given simultaneously. However, the additional cytotoxicity produced when cisplatin followed the hypoxic exposure to TPZ was not seen in the cross-link repair-deficient UV41 cells, whereas it was present in the wild-type AA8 cells. These data, therefore, suggest that prior exposure of cells to TPZ under hypoxic conditions may partially inhibit the repair of DNA cross-links. One caveat to these data, however, is that in order to have comparable cell survival of the UV41 and AA8 cells to cisplatin and TPZ alone, it was necessary to give much lower doses of the two drugs with the UV41 cells (60-fold lower for cisplatin and threefold lower for TPZ compared to the doses used for the AA8 cells).

To more directly test the effect of TPZ on interstrand cross-links, we next measured DNA interstrand cross-links, using alkaline filter elution, following cisplatin exposure with or without prior treatment to TPZ under hypoxia. In these experiments, NIH 3T3 cells were treated with cisplatin (50 μM for 1 h) either alone, or 30 min after the end of a 1-h exposure to TPZ (20 μM under hypoxia). The cells treated with cisplatin alone were given a 1-h treatment of hypoxia starting 1.5 h prior to the cisplatin exposure. Figure 4 shows the alkaline elution data at 48 and 72 h after the cisplatin treatment, and the calculation of the DNA interstrand cross-link factor from 0 to 72 h following cisplatin exposure. The data show that the initial level of DNA interstrand cross-links is similar for the two groups, but the cells treated with TPZ under hypoxia prior to cisplatin have less repair of the cross-links after the peak at approximately 12 h after exposure compared with cells treated with cisplatin alone. The area under the curve of cross-links as a function of time (which has been shown to correlate with cytotoxicity; Hansson et al, 1987) for the TPZ + cisplatin group is 2.8 times greater than that for the cisplatin only group. This is to be compared with a DMF of 3.5–4.0 for cell killing (Figure 2). Considering interexperimental variability and the very different nature of the assays involved, these data support the conclusion that most, if not all, of the potentiation of cisplatin cell kill by TPZ can be accounted for by an increased incidence of DNA interstrand cross-links.

### Oxygen dependence of cisplatin sensitization by TPZ

In all of the experiments described above, the TPZ exposure was given under conditions of extreme hypoxia (approximately 0.02%

 © 1999 Cancer Research Campaign

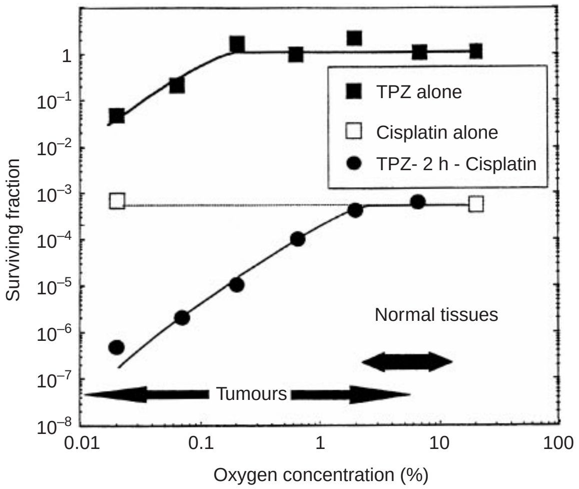

**Figure 5** The effect of varying oxygen concentrations on the cytotoxicity of NIH 3T3 cells treated with TPZ (1.5 h) alone (25 μM), with cisplatin alone (20 μM), or with TPZ followed 2 h later with cisplatin. The data are pooled from two experiments. Also shown is the range of oxygen concentrations typical of normal tissues and of tumours oxygen measured in the medium). In many control experiments (data not shown), we found no interaction when the TPZ was given under aerobic conditions (20% oxygen). Since the median oxygen concentration in tumours spans a wide range from approximately 0.1 to 4% (Lartigau et al, 1993; Nordsmark et al, 1994; Vaupel and Hockel, 1995), we tested the interaction between TPZ and cisplatin as a function of oxygen concentration. In this experiment, NIH 3T3 cells were exposed under varying oxygen concentrations for 1.5 h either to 25 μM TPZ or to a combination of TPZ with 20 μM cisplatin started 1 h after the end of the TPZ exposure. The results (Figure 5) show that the interaction between the two drugs starts to occur at oxygen concentrations less than 1% and before there is any cytotoxicity by the TPZ alone. At very low oxygen concentrations, the additional cell kill produced by the combination of TPZ and cisplatin over that of cisplatin alone is a result both of cell killing by TPZ and of the interaction between the two drugs. Also shown on Figure 5 are the typical ranges of oxygen concentrations of cells in tumours and subcutaneous normal tissues.

### DISCUSSION

The purpose of the present study was to investigate the mechanism by which TPZ potentiates the cytotoxicity of cisplatin. We have previously shown that there is a tumour-specific synergistic interaction between these two agents in transplanted mouse tumours when TPZ is given prior to, but not at the same time as, cisplatin (Dorie and Brown, 1993). Although an interaction between TPZ and other anticancer drugs has been reported (Langmuir et al, 1994; Siemann, 1996; Dorie and Brown, 1997), none is as dramatic as with platinum-containing drugs and may result from complementary cytotoxicity within the tumour and/or may not lead to a therapeutic gain (Siemann, 1996). The tumour specificity of the interaction between TPZ and cisplatin has led to clinical trials of the combination of the two drugs, which have shown that TPZ can produce a therapeutic gain by potentiating the anti-tumour efficacy of cisplatin without changing its systemic toxicity (Treat et al, 1998; von Pawel and von Roemeling, 1998). Our experiments in the present study show that the interaction between the two drugs depends on the TPZ exposure being under oxygen concentrations of less than 1%, and that it can be demonstrated at the cellular level. The fact that the characteristics and timing of the interaction (i.e. additive when the two drugs are given simultaneously, but synergistic when TPZ precedes cisplatin) are similar for both the in vitro and in vivo exposures suggests that most, if not all, of the in vivo synergy is the consequence of a cellular interaction between the two drugs. This was by no means obvious since it has been shown by several investigators that hypoxic cells in tumours are preferentially spared from cisplatin cell kill (Grau and Overgaard, 1988), and complementary cytotoxicity between TPZ and cisplatin could have produced an apparently synergistic interaction. However, complementary cell kill could still account for part of the interaction in vivo, depending on the dose of TPZ administered and the sensitivity of the tumour cells to TPZ-induced cell kill. It could also be responsible for the (lesser) interaction between TPZ and other anticancer drugs (Dorie and Brown, 1997).

Our data on the oxygen dependence of the interaction between the two drugs can account for the tumour specificity of cisplatin potentiation by TPZ. We show (Figure 5) that at oxygen concentrations typical of normal tissues, there is no interaction between TPZ and cisplatin, whereas at lower oxygen concentrations, which occur commonly in tumours, TPZ potentiates the cytotoxicity of cisplatin. It seems reasonable, therefore, to conclude that the tumour specific potentiation of cisplatin cytotoxicity seen in both preclinical and clinical studies is the result of the lower oxygen levels present in solid tumours. The presence of such hypoxic regions has been demonstrated in all human solid tumours so far studied, including brain (Cruickshank et al, 1994), head and neck (Nordsmark et al, 1994; Adam et al, 1998), lung (Urtasun et al, 1986), breast (Vaupel et al, 1991), cervix (Hockel et al, 1991), melanoma (Lartigau et al, 1997), anal canal (Mattern et al, 1996) and soft tissue sarcomas (Brizel et al, 1994). All of these data show that the median oxygen concentration of human solid tumours is highly heterogeneous, but that the vast majority of tumours have median oxygen levels lower than those of normal tissues.

It should also be pointed out that the present data demonstrate a significant potentiation of cisplatin cytotoxicity at oxygen levels considerably higher than those normally associated with the 'hypoxic fraction' of tumours, which usually means cells at maximum radiation resistance, or below 0.1–0.2% oxygen (Koch et al, 1984). It has previously been shown that TPZ has a different oxygen dependency for cytotoxicity compared to other bioreductive drugs of the nitroimidazole or quinone antibiotic class in that it produces cytotoxicity at higher oxygen concentrations (Koch, 1993). We show here that significant potentiation of cisplatin cytotoxicity occurs at oxygen concentrations at which TPZ alone is not cytotoxic (Figure 5). It seems reasonable, therefore, to suppose that in many tumours most, if not all, of the cells may be sensitized to cisplatin cell kill by TPZ. Indeed, in elegant studies of oxygen levels in human tumour xenografts, Helmlinger and colleagues (Helmlinger et al, 1997) showed that the mean oxygen level adjacent to capillaries is 15 mmHg, or approximately 2% oxygen. Given our data showing cisplatin potentiation by TPZ beginning at 1–2% oxygen (Figure 5), this could readily account for the published data showing potentiation of cisplatin cytotoxicity by TPZ in all the cells of xenografted human tumours, including those closest to the blood vessels (Durand, 1994).

The present data provide further insight into the mechanism by which hypoxic exposure to TPZ potentiates cisplatin cytotoxicity. First, we show that this interaction is absent in CHO UV41 cells,

© 1999 Cancer Research Campaign which are deficient in the ERCC4 protein and in the repair of DNA interstrand cross-links (Andersson et al, 1996). These data implicate ERCC4, or its partner ERCC1, or other proteins involved in the repair of DNA interstrand cross-links, as involved in the mechanism of the potentiation. We obtained direct evidence for this by measuring DNA interstrand cross-links in cells following cisplatin treatment with or without prior exposure to TPZ under hypoxia. The fact that we saw similar levels of initial DNA cross-links, but higher levels following a repair period suggests that TPZ exposure either inhibits cross-link repair or the repair of cisplatin monoadducts prior to their formation of interstrand cross-links.

At present we can only speculate on the possible mechanism by which TPZ could inhibit the repair of DNA interstrand cross-links produced by cisplatin. However, our recent finding that all of the DNA damage produced by TPZ occurs by metabolism of the drug within the nucleus (Evans et al, 1998), and that protein fractions associated with the nuclear matrix are highly active in TPZ metabolism (Delahoussaye et al, 1997), suggests that TPZ damage could be preferentially localized to DNA closely associated with the nuclear matrix. Since the nuclear matrix is the site for DNA replication (Berezney and Coffey, 1975) and transcription (Cook, 1989), TPZ damage could have an effect on these processes. We have in fact observed a large inhibition of both DNA replication and transcription by TPZ even at minimally cytotoxic concentrations (KB Peters et al, unpublished observations). We suggest therefore that sensitization of cells to cisplatin could be the result of an inhibition by TPZ of transcription of genes involved in the repair of DNA cross-links following cisplatin exposure. In this regard it has recently been reported that cisplatin exposure of ovarian cancer cells increases transcription and protein levels of ERCC1 (Li et al, 1998). As message levels of this gene have been reported to correlate with cisplatin sensitivity (Dabholkar et al, 1992), it seems reasonable to speculate that inhibition by TPZ of enhanced transcription of ERCC1 would sensitize the cells to cisplatin cytotoxicity. Experiments to test this hypothesis are currently underway.

In conclusion, we show that the tumour-specific potentiation of cisplatin cell kill by TPZ can be accounted for by a cellular interaction between the two drugs that appears to involve a diminished, or delayed, repair of cisplatin-induced interstrand cross-links. We further show that this interaction can occur at non-toxic concentrations of TPZ, but requires the TPZ exposure to be under oxygen concentrations lower than are those of normal tissues, but typical of those in solid tumours. These data provide mechanistic insight into the preclinical and clinical data showing that TPZ can produce a therapeutic gain when added to cisplatin in the treatment of solid tumours.

### ACKNOWLEDGEMENTS

This work was supported by USPHS grant CA 67166 (to JMB), and by a National Cancer Institute of Canada Junior Research Fellowship #6453 (to BGW).

### REFERENCES

Adam M, Gabalski EC, Oehlert JW, Bloch DA, Brown JM, Elsaid AA, Pinto HA and Terris DJ (1999) Tissue oxygen distribution in head and neck patients. *Head Neck* **21**: 146–153

Andersson BS, Sadeghi T, Siciliano MJ, Legerski R and Murray D (1996) Nucleotide excision repair genes as determinants of cellular sensitivity to cyclophosphamide analogs. *Cancer Chemother Pharmacol* **38**: 406–416

Berezney R and Coffey DS (1975) Nuclear protein matrix: association with newly synthesized DNA. *Science* **189**: 291–293

Brizel DM, Rosner GL, Harrelson J, Prosnitz LR and Dewhirst MW (1994) Pretreatment oxygenation profiles of human soft tissue sarcomas. *Int J Radiat Oncol Biol Phys* **30**: 635–642

Brizel DM, Sibley GS, Prosnitz LR, Scher RL and Dewhirst MW (1997) Tumor hypoxia adversely affects the prognosis of carcinoma of the head and neck. *Int I Radiat Oncol Biol Phys* **38**: 285–289

Brown JM (1993) SR 4233 (tirapazamine): a new anticancer drug exploiting hypoxia in solid tumours. *Br J Cancer* **67**: 1163–1170

Brown JM and Lemmon MJ (1990) Potentiation by the hypoxic cytotoxin SR 4233 of cell killing produced by fractionated irradiation of mouse tumors. *Cancer Res* **50**: 7745–7749

Brown JM and Lemmon MJ (1991) Tumor hypoxia can be exploited to preferentially sensitize tumors to fractionated irradiation. *Int J Radiat Oncol Biol Phys* **20**: 457–461

Cook PR (1989) The nucleoskeleton and the topology of transcription. *Eur J Biochem* **185**: 487–501

Cruickshank GS, Rampling RP and Cowans W (1994) Direct measurement of the $PO_2$ distribution in human malignant brain tumours. *Adv Exp Med Biol* **345**: 465–470

Dabholkar M, Bostick-Bruton F, Weber C, Bohr VA, Egwuagu C and Reed E (1992) ERCC1 and ERCC2 expression in malignant tissues from ovarian cancer patients. *J Natl Cancer Inst* **84**: 1512–1517

Damia G, Imperatori L, Stefanini M and D'Incalci M (1996) Sensitivity of CHO mutant cell lines with specific defects in nucleotide excision repair to different anti-cancer agents. *Int J Cancer* **66**: 779–783

Delahoussaye YM, Wouters BG, Evans JE and Brown JM (1997) Intranuclear metabolism of tirapazamine by matrix-associated reductases. *Proc Am Assoc Cancer Res* **38**: 163 (abstr. # 1095)

Dorie MJ and Brown JM (1993) Tumor-specific, schedule-dependent interaction between tirapazamine (SR 4233) and cisplatin. *Cancer Res* **53**: 4633–4636

Dorie MJ and Brown JM (1997) Modification of the antitumor activity of chemotherapeutic drugs by the hypoxic cytotoxic agent tirapazamine. *Cancer Chemother Pharmacol* **39**: 361–366

Durand RE (1994) The influence of microenvironmental factors during cancer therapy. *In vivo* **8**: 691–702

Evans JE, Yudoh K, Delahoussaye YM and Brown JM (1998) Tirapazamine is metabolized to its DNA damaging radical by intranuclear enzymes. *Cancer Res* **58**: 2098–2101

Ewig RA and Kohn KW (1978) DNA-protein cross-linking and DNA interstrand cross-linking by haloethylnitrosoureas in L1210 cells. *Cancer Res* **38**: 3197–3203

Gatenby RA, Kessler HB, Rosenblum JS, Coia LR, Moldofsky PJ and Hartz WH (1988) Oxygen distribution in squamous cell carcinoma metastases and its relationship to outcome of radiation therapy. *Int J Radiat Oncol Biol Phys* **14**: 831–838

Graham MA, Senan S, Robin H, Eckhardt N, Lendrem D, Hincks J, Greenslade D, Rampling R, Kaye SB, von Roemeling R and Workman P (1997) Pharmacokinetics of the hypoxic cell cytotoxic agent tirapazamine and its major bioreductive metabolites in mice and humans: retrospective analysis of a pharmacokinetically guided dose escalation strategy in a phase I trial. *Cancer Chemother Pharmacokin* **40**: 1–10

Grau C and Overgaard J (1988) Effect of cancer chemotherapy on the hypoxic fraction of a solid tumor measured using a local tumor control assay. *Radiother Oncol* **13**: 301–309

Hansson J, Lewensohn R, Ringborg U and Nilsson B (1987) Formation and removal of DNA cross-links induced by melphalan and nitrogen mustard in relation to drug-induced cytotoxicity in human melanoma cells. *Cancer Res* **47**: 2631–2637

Helmlinger G, Yuan F, Dellian M and Jain RK (1997) Interstitial pH and $pO_2$ gradients in solid tumors in vivo: high-resolution measurements reveal a lack of correlation. *Nat Med* **3**: 177–182

Hockel M, Schlenger K, Knoop C and Vaupel P (1991) Oxygenation of carcinomas of the uterine cervix: evaluation by computerized $O_2$ tension measurements. *Cancer Res* **51**: 6098–6102

Jones NJ, Stewart SA and Thompson LH (1990) Biochemical and genetic analysis of the Chinese hamster mutants irs1 and irs2 and their comparison to cultured ataxia telangiectasia cells. *Mutagenesis* **5**: 15–23

Kim IH and Brown JM (1994) Reoxygenation and rehypoxiation in the SCCVII mouse tumor. *Int J Radiat Oncol Biol Phys* **29**: 493–497

Koch CJ (1993) Unusual oxygen concentration dependence of toxicity of SR-4233, a hypoxic cell toxin. *Cancer Res* **53**: 3992–3997

 © 1999 Cancer Research Campaign

Miller VA, Ng KK, Grant SC, Kindler H, Pizzo B, Heelan RT, von Roemeling R and Kris MG (1997) Phase II study of the combination of the novel bioreductive agent, tirapazamine, with cisplatin in patients with advanced non-small-cell lung cancer. *Ann Oncol* **8**: 1269–1271

Nordsmark M, Bentzen SM and Overgaard J (1994) Measurement of human tumour oxygenation status by a polarographic needle electrode. *Acta Oncol* **33**: 383–389

Nordsmark M, Overgaard M and Overgaard J (1996) Pretreatment oxygenation predicts radiation response in advanced squamous cell carcinoma of the head and neck. *Radiother Oncol* **41**: 31–40

Rodriguez GI, Valdivieso M, Von Hoff DD, Kraut M, Burris HA, Eckardt JR, Lockwood G, Kennedy H and von Roemeling R (1996) A phase I/II trial of the combination of tirapazamine and cisplatin in patients with non-small cell lung cancer (NSCLC). *Proc Am Soc Clin Oncol* **15**: 382 (abstract)

Siemann DW (1996) The in situ tumour response to combinations of cyclophosphamide and tirapazamine. *Br J Cancer* **74**: S65–69

Siemann DW and Hinchman CA (1998) Potentiation of cisplatin activity by the bioreductive agent tirapazamine. *Radiother Oncol* **47**: 215–220

Treat J, Haynes B, Johnson E, Belani C, Greenberg R, Rodriquez R, Drobbins P, Miller WJ, Meehan L and von Roemeling R (1997) Tirapazamine with cisplatin: a phase II trial in advanced stage non-small cell lung cancer (NSCLC). *Proc Am Soc Clin Oncol* **16**: 1633 (abstract)

Treat J, Johnson E, Langer C, Belani C, Haynes B, Greenberg R, Rodriquez R, Drobins P, Miller W, Jr Meehan L, McKeon A, Devin J, von Roemeling R and Viallet J (1998) Tirapazamine with cisplatin in patients with advanced non-small-cell lung cancer: a phase II study. *J Clin Oncol* **16**: 3524–3527

Urtasun RC, Chapman JD, Raleigh JA, Franko AJ and Koch CJ (1986) Binding of 3H-misonidazole to solid human tumors as a measure of tumor hypoxia. *Int J Radiat Oncol Biol Phys* **12**: 1263–1267

Vaupel PW and Hockel M (1995) Oxygenation status of human tumors: a reappraisal using computerized $pO_2$ histography. In *Tumor Oxygenation*, Vaupel PW, Kelleher DK and Gunderoth M (ed) pp. 219–232. Gustav Fischer Verlag: Stuttgart

Vaupel PW, Schlenger K, Knoop C and Hockel M (1991) Oxygenation of human tumors: evaluation of tissue oxygen distribution in breast cancers by computerized $O_2$ tension measurements. *Cancer Res* **51**: 3316–3322

von Pawel J and von Roemeling R (1998) Survival benefit from tirazone (tirapazamine) and cisplatin in advanced non-small cell lung cancer (NSCLC) patients: final results from the international phase III CATAPULT 1 trial. *Proc Am Soc Clin Oncol* **17**: 454a (Abstract)

Zeman EM, Brown JM, Lemmon MJ, Hirst VK and Lee WW (1986) SR-4233: a new bioreductive agent with high selective toxicity for hypoxic mammalian cells. *Int J Radiat Oncol Biol Phys* **12**: 1239–1242

*© 1999 Cancer Research Campaign*
